# Emergency Surgery Score as a Predictor of Post-operative Outcomes in Non-traumatic Emergency Laparotomy: A Prospective Observational Study

**DOI:** 10.7759/cureus.90079

**Published:** 2025-08-14

**Authors:** Sudhir K Singh, Sanjay Katragadda, Summi Karn, Afsar Ali, Adarsh Jha, Ashish Mishra, Navin Kumar, Farhanul Huda, Somprakas Basu

**Affiliations:** 1 General Surgery, All India Institute of Medical Sciences, Rishikesh, Rishikesh, IND

**Keywords:** 30-day mortality, emergency abdominal surgery, emergency exploratory laparotomy, emergency surgery score, length of hospital stay, postoperative complications, surgical outcome

## Abstract

Introduction

Most patients with acute abdominal conditions present with a unique set of problems because of acute physiological derangements, lack of optimization, and associated comorbidities. Emergency laparotomy in such patients is quite a morbid procedure. Predicting the surgical outcome and taking measures in advance to improve patient outcomes following emergency laparotomy is of utmost importance. Simultaneously, it can also help in preoperative counseling and shared decision-making for patient management. This study was designed to investigate the potential use of Emergency Surgery Score (ESS) in predicting outcomes among non-traumatic emergency laparotomy cases.

Material and methods

This prospective observational study was conducted at the Department of General Surgery at a Tertiary Care Centre in India. All patients above 18 years of age undergoing nontraumatic emergency laparotomy were included in the study. All variables related to the three broad categories of ESS were recorded. Each ESS variable was scored accordingly, except for the variable "White race," which was scored zero by default. Surgical outcome was recorded in terms of length of hospital stay, length of ICU stay, 30-day mortality rate, 30-day postoperative complications, and 30-day readmission.

Results

A total of 163 patients were included in this study. All the variables related to the ESS and surgical outcomes were recorded and analyzed. ESS correlated positively with the length of hospital and ICU stay among the survivors (rho=0.51 and rho=0.53, respectively). For a one-unit rise in ESS, the length of hospital stay and the length of ICU stay increased by 1.8 days and 0.71 days, respectively. Using Receiver Operating Characteristic (ROC) analysis, the ESS demonstrated a strong correlation with both the 30-day mortality rate and 30-day complications (Area under the Receiver Operating Characteristic Curve (AUROC) values of 0.895 and 0.875, respectively).

Conclusion

Our study's findings underscore the potential of ESS as a powerful predictor of postoperative outcomes in emergency laparotomies. By providing an objective measure for outcome comparison, ESS can significantly enhance preoperative patient counseling and decision-making.

## Introduction

Emergency surgeries comprise a significant part of the operative cases in all surgical specialties. While there is no universally accepted definition of emergency surgery, it is generally understood as a procedure that must be performed as soon as possible following the diagnosis or the onset of related preoperative symptoms, where any delay could potentially endanger the patient’s well-being and outcome [1]. To underscore the burden of emergency surgery, a retrospective review of data from the 2008-2011 National Inpatient Sample in the United States demonstrated that more than half a million patients undergo urgent or emergent general surgery operations annually [2].

Emergency surgeries carry a high risk of postoperative complications, including mortality, when compared to an elective surgery. A retrospective study by Havens et al. concluded that the mortality rate was significantly higher in emergency surgeries compared to elective surgeries (12% vs. 2%) [3]. Sorenson et al. observed that patients undergoing emergency open gastrointestinal surgery have a five-fold higher risk of 30-day mortality compared to elective operations [4]. Due to the high rate of postoperative complications, emergency surgeries have a higher requirement for ICU resources as well [5]. Emergency surgery is an independent risk factor for postoperative complications and death, even when controlled for preoperative variables and procedure type [6].

Given the poor postoperative outcomes in emergency surgery, it is imperative to identify and detect postoperative complications early. The outcome can be predicted through an accurate scoring system, which can significantly improve patient care. While the American Society of Anesthesiologists (ASA) score, Physiological and Operative Severity Score for the enUmeration of Mortality and Morbidity (POSSUM), and Surgical Risk Scale have been developed, none of them is specific to emergency surgery [7]. To address this gap, Sangji et al. developed the Emergency Surgery Score (ESS), a new scoring system comprising 22 specific parameters that provide a comprehensive assessment of patient risk and predict outcomes of emergency procedures [8].

Despite numerous studies on the ESS and its practical application, there is a noticeable dearth of research on ESS in the Indian context. The ESS, a relatively novel scoring tool, is not widely understood in terms of its practical application. The primary objective of this study is to determine the effectiveness of the ESS in predicting the postoperative outcomes of nontraumatic emergency laparotomies in an Indian setting.

## Materials and methods

This prospective observational study was conducted in the Department of General Surgery at All India Institute of Medical Sciences, Rishikesh, Rishikesh, a tertiary care center in India, over a period of 18 months from June 2022 to December 2023. All patients aged 18 years or older undergoing emergency laparotomy were included in the study. Patients with traumatic abdominal emergencies were excluded from the study. All variables related to three broad categories of ESS (i.e., demographics, comorbidities, and biochemical investigations) were recorded [8].

Each variable was scored accordingly, except for White race, which was assigned a score of zero for every patient. The ESS for each participant was computed and recorded. Decisions regarding surgical technique, postoperative care, discharge, and readmission were left solely to the treatment team, as per institutional guidelines. Every patient was assessed and followed to record the 30-day mortality rate, 30-day postoperative complications as per the Clavien-Dindo classification [9], length of hospital stay, length of ICU stay, and 30-day readmission. This study was purely observational and had no implications for clinical care throughout its duration.

For statistical analysis, categorical variables were described in terms of frequency and proportion, while continuous variables were described as mean ± standard deviation (SD) or median with interquartile range (IQR) as applicable. For inferential statistical analysis, the association between qualitative variables was determined by the Chi-square test or Fisher’s exact test. The means in the two groups were compared using the Student’s t-test or the Wilcoxon Mann-Whitney U test. Analysis of variance (ANOVA) or Kruskal-Wallis tests were used to compare means in more than two groups as appropriate. To assess correlation statistics, Pearson’s correlation, Point-Biserial Correlation, or Spearman’s correlation coefficient was used, depending on the distribution of data. The discriminant power of the score was evaluated by using Receiver Operating Characteristic (ROC) and Area under the Receiver Operating Characteristic Curve (AUROC). A P-value less than 0.05 was considered statistically significant.

## Results

A total of 163 patients were included in this study, and all the variables related to the ESS score were recorded and analyzed. The mean (SD) age of presentation of the patients was 42.70 (15.25) years, with a median (IQR) of 42 (30-54) years. The minimum and maximum ages of presentation were 19 years and 82 years, respectively. Among the subjects, 112 (69%) were male and 51 (31%) were female. Age was normally distributed among both genders, and no significant difference was found between the groups in terms of age (years) (t=0.778, p=0.439). Patients presented with a wide range of disease spectra, including hollow viscus perforation peritonitis (n=89; 55%), acute intestinal obstruction (n=57; 35%), iatrogenic bile duct injury with diffuse biliary peritonitis, acute mesenteric ischemia, and strangulated hernia.

Upon evaluating the demographic variables related to ESS, it was noted that 25 (15.3%) patients were 60 years or older. None of the study population belonged to the White race; therefore, it was scored as zero by default. Seventy-three patients were transferred from another acute care center (i.e., from outside), and nine patients were transferred from an acute inpatient facility.

**Figure 1:**
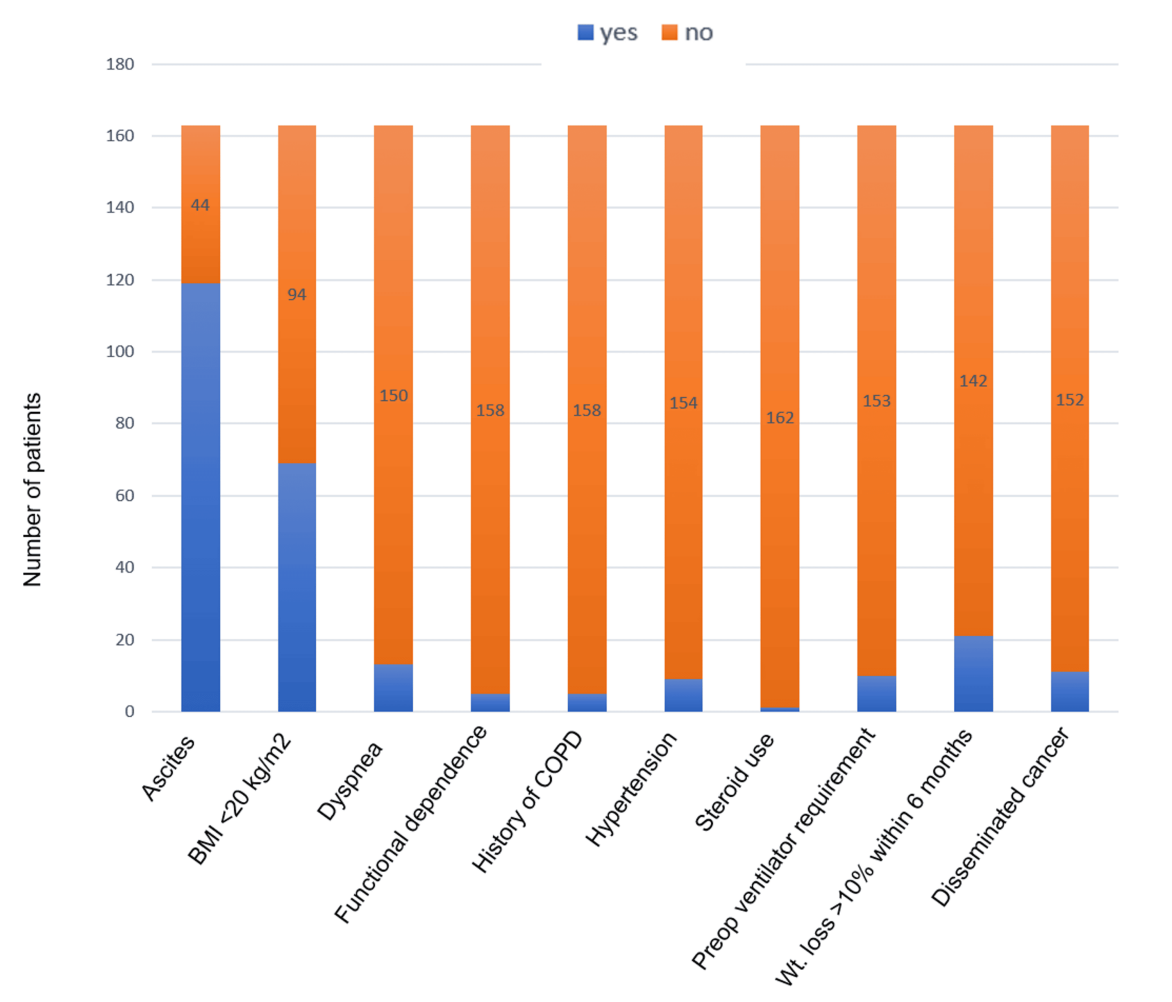
Distribution of comorbidities in the study population (n=163) COPD: Chronic Obstructive Pulmonary Disease; WL: Weight loss

Analysis of the distribution of various comorbidities revealed that a significant proportion of the population presented with ascites and poor nutritional status. The rest of the comorbidities were present only in a small proportion of the population. The comorbidities with higher weightage as per ESS include ventilator requirement 48 hours before surgery and disseminated cancer, which were present in 10 (6%) and 11 (6.7%) patients, respectively (Figure 1).

**Figure 2:**
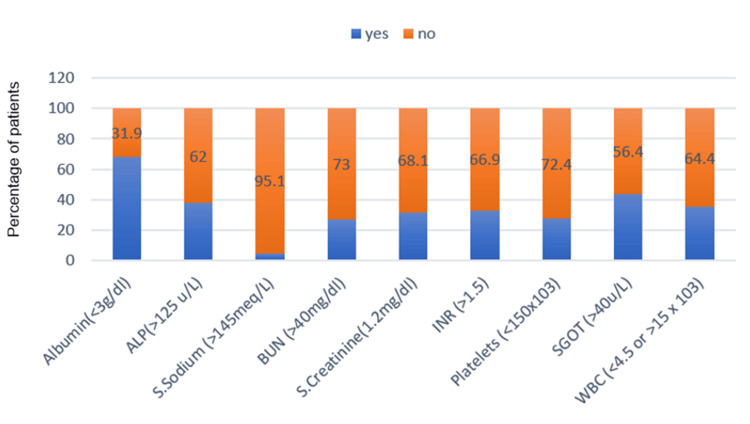
Distribution of biochemical parameters in the study population (n=163) ALP: alkaline phosphatase; BUN: blood urea nitrogen; INR: international normalized ratio; SGOT: serum glutamic-oxaloacetic transaminase; WBC: white blood cell. Yes and No indicate whether the mentioned specific biochemical parameter cutoff value is present or absent, respectively.

Among the biochemical parameters, albumin levels were less than 3 g/dL in 111 (68%) patients. Almost all the other parameters were deranged in quite a significant proportion of the study population, except for serum sodium (Figure 2).

On analysis, the mean (SD) and median (IQR) of ESS were 6.13 (3.61) and 5.00 (3-8), respectively. The ESS ranged from 0 to 19. The data was positively skewed (skewness=0.88) and not normally distributed. An analysis of surgical outcomes was conducted for all the study participants, which included length of hospital stay, length of ICU stays, 30-day postoperative complications, 30-day readmission, and 30-day mortality. The mean (SD) of the length of hospital stay and ICU stay among survivors was 13.44 (11.61) and 2.81 (5.50) days, respectively. A total of 48 patients died within 30 days of being operated, leading to a mortality rate of 29%, and only five patients were readmitted within 30 days. Out of the 163 patients, only 34 had no complications, whereas the rest of the patients had at least a Clavien-Dindo grade 1 complication (Table 1).

**Table 1:** Distribution of the study population as per grading of the Clavien-Dindo classification [9]

Clavien-Dindo grade	Frequency	Percentage	95% CI
0	34	20.90%	15.1% - 28.1%
1	27	16.60%	11.4% - 23.4%
2	21	12.90%	8.3% - 19.2%
3a	26	16.00%	10.9% - 22.7%
3b	4	2.50%	0.8% - 6.6%
4a	2	1.20%	0.2% - 4.8%
4b	1	0.60%	0.0% - 3.9%
5	48	29.40%	22.7% - 37.2%

**Figure 3:**
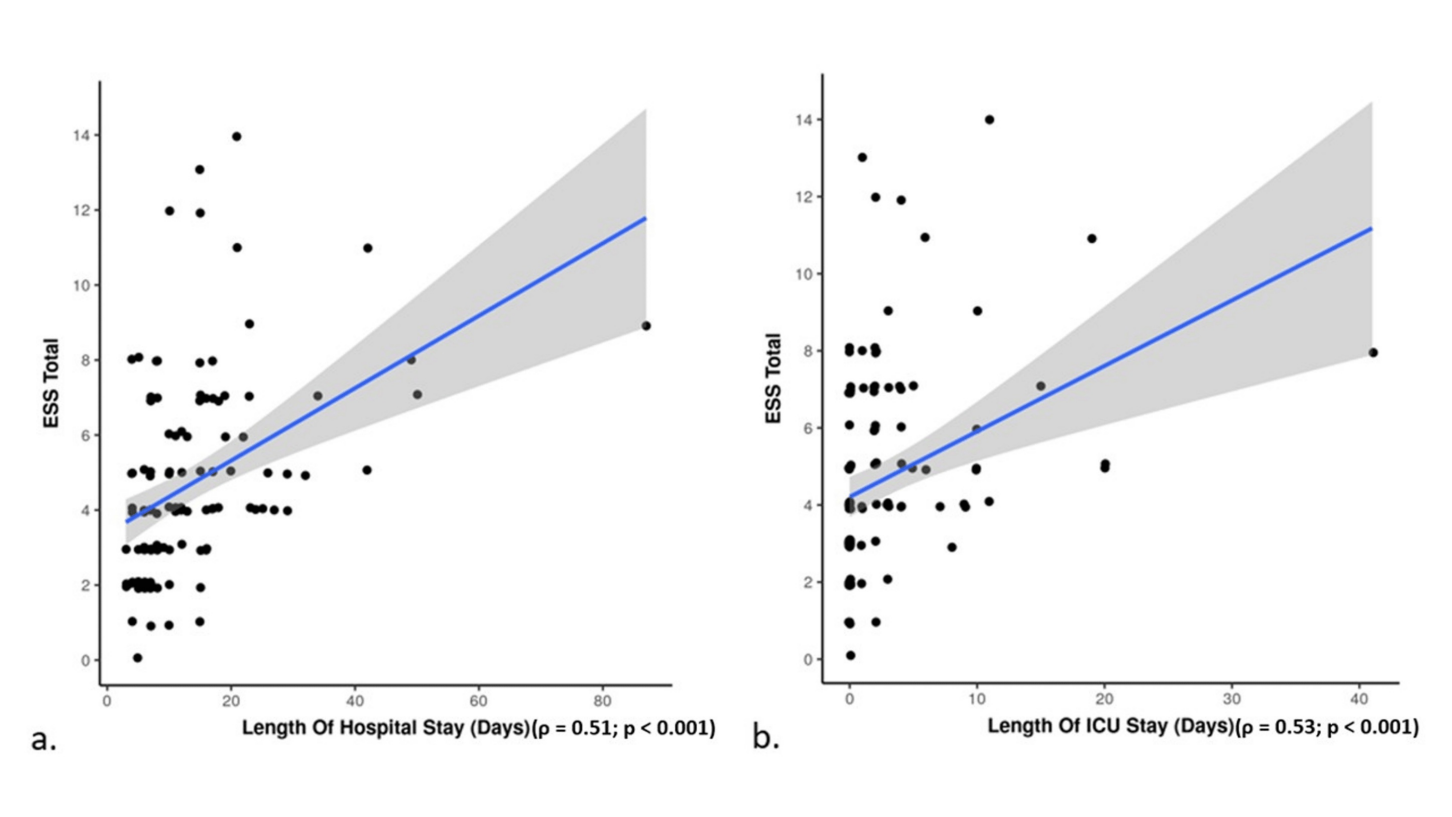
Scatterplot graph showing association between ESS and length of (a) hospital stay and (b) ICU stay Spearman Correlation (ρ); ESS: Emergency Surgery Score

The Spearman correlation was used to observe the correlation of ESS with the length of hospital stay and the length of ICU stay among survivors. The coefficient (ρ) was 0.51 (p<0.001) and 0.53 (p<0.001) for the length of hospital stay and the length of ICU stay, respectively, indicating a moderate positive correlation. This correlation is depicted with the help of a scatterplot (Figure 3).

The subgroups of 30-day mortality and complications were compared using the Wilcoxon-Mann-Whitney U Test. The two groups of 30-day mortality differed significantly in terms of ESS (W=4943.000, p<0.001), with the 30-day mortality group demonstrating a higher median ESS (Table 2).

**Table 2:** Showing the association of ESS with 30-day mortality ESS: Emergency Surgery Score

ESS total	30-day mortality		Wilcoxon-Mann-Whitney U test	
	Yes	No	W	p value
Mean (SD)	9.60 (3.17)	4.69 (2.69)	4943	<0.001
Median (IQR)	9 (7-11.25)	4 (3-6)		
Min - Max	5-19	0-14		

Similarly, the two groups of complications differed significantly in terms of ESS (W=3837.500, p<0.001), with the 30-day complication group showing a higher median ESS. The strength of association was determined using the Point-Biserial Correlation, which yielded values of 0.62 and 0.46 for 30-day mortality and complications, respectively, indicating a large effect size (Table 3).

**Table 3:** Showing the association of ESS with complications ESS: Emergency Surgery Score

ESS total	Complications		Wilcoxon-Mann-Whitney U test	
	Yes	No	W	p value
Mean (SD)	6.99 (3.50)	2.88 (1.68)	3837.5	<0.001
Median (IQR)	7 (4-9)	2 (2-3.75)		
Min - Max	1-19	0-8		

The two subgroups of the variable of 30-day readmission groups were also compared to find the association with ESS but no significant difference was seen (W=392.000, p=0.981) (Table 4).

**Table 4:** Showing the association of ESS with 30-day readmission ESS: Emergency Surgery Score

ESS	30-day readmission		Wilcoxon-Mann-Whitney U test	
	Yes	No	W	p value
Mean (SD)	5.60 (1.52)	6.15 (3.66)	392	0.981
Median (IQR)	6 (4-7)	5 (3-8)		
Min – Max	4-7	0-19		

The Clavien-Dindo complication groups were compared using the Kruskal-Wallis Test, as ESS was not normally distributed in the various subgroups. The groups differed significantly in terms of ESS (χ2=87.686, p<0.001), with group 4a having a higher median ESS. The strength of association was computed using Kendall's Tau, which was 0.58, implying a large effect size (Table 3).

**Table 5:** Association between the ESS and Clavien-Dindo grades ESS: Emergency Surgery Score

ESS	Clavien-Dindo grade								Kruskal Wallis test	
	0	1	2	3a	3b	4a	4b	5	χ2	p value
Mean (SD)	2.88 (1.68)	4.74 (2.35)	5.38 (2.67)	6.04 (2.63)	4.25 (1.71)	9.50 (6.36)	7.00 (NA)	9.60 (3.17)	87.686	<0.001
Median (IQR)	2 (2-3.75)	4 (3-6.5)	4 (4-7)	5.5 (4-7)	4.5 (3.5-5.25)	9.5 (7.25-11.75)	7 (7-7)	9 (7-11.25)		
Min - Max	0-8	1-11	1-12	2-13	2-6	5-14	7-7	5-19		

**Figure 4:**
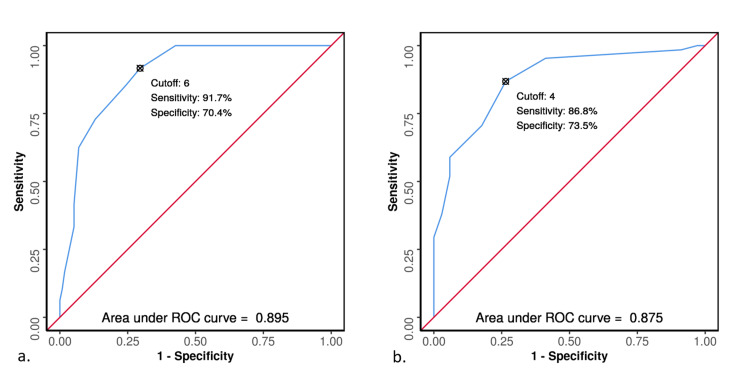
ROC curve analysis showing diagnostic performance of ESS in predicting (a) 30-day mortality and (b) 30-day complications ROC: Receiver Operating Curve; ESS: Emergency Surgery Score

To identify the performance of ESS in predicting 30-day mortality and 30-day complications, the AUROC was analyzed. The AUROC for ESS predicting 30-day mortality was 0.895 (95% CI: 0.848-0.942; p<0.001), indicating good diagnostic performance. At a cut-off of ESS total ≥6, it predicted 30-day mortality with a sensitivity of 92% and a specificity of 70%. Similarly, the AUROC for ESS predicting complications was 0.875 (95% CI: 0.811-0.938; p<0.001), demonstrating good diagnostic performance. At an ESS cut-off of four, the sensitivity and specificity in predicting complications were 87% and 74%, respectively (Figure 4).

## Discussion

Upon analyzing the data of 163 patients recruited for this study, we found that the demographics of the study population differed markedly from those in international statistics. The mean age of patients undergoing laparotomy was 42.70 years, which is in stark contrast to the 60.5 years mean age of patients undergoing emergency laparotomy in the prospective study done by Kaafarani et al. [10]. However, Manoj et al. studied the demography of the patients undergoing emergency laparotomies in a tertiary health center in India and noted the mean age of presentation to be 42.99 years [11]. The reason for the presentation of younger age groups in our setting to emergency rooms can be because of the high rate of smoking and abuse of over-the-counter analgesic medications, the high prevalence of H. pylori infection, and the delay in presentation to health centers [12,13]. Approximately 70% of the study population was made up of male patients, whereas 50% of the male population was reported in studies by Kaafarani et al. and Nandan et al. [10,14]. In the Indian setting, Manoj et al. reported similar statistics of around 23% of female patients presenting to the emergency department and eventually undergoing emergency laparotomy [11].

The common indications for emergency laparotomy in this study population were hollow viscus perforation with peritonitis (55%) and acute intestinal obstruction (35%). Other less common indications for emergency laparotomy reported were strangulated hernia, iatrogenic bile duct injury with diffuse biliary peritonitis, and acute mesenteric ischemia. Studies elsewhere report a similar trend, with the most common indication of non-traumatic laparotomy being intestinal perforation, followed by intestinal obstruction [12,14,15].

Upon tracing the referral pattern, approximately 45% of cases were referred by local practitioners, and 5% were referred from centers with an inpatient facility. Most of these patients were referred due to comorbidities and the need for ICU care. A significant difference in 30-day mortality was observed between the two patient groups in this study: those referred and those who presented to the emergency room without referral. Our study population had a 30-day mortality rate of 29.4%, which was higher compared to the mortality rates in the Western population, which ranged from 14% to 18%. However, medical comorbidities were less prevalent than in the Western study population [3,16,17]. The referred patients contributed to 64% of the total mortality, and they were high-risk cases, which might be a probable cause of the high rate of mortality compared to the Western study population. The high 30-day mortality rate in our study population could be attributed to the high prevalence of comorbidities and the delay in presentation to health centers.

The mean and median lengths of hospital stay in survivors were 13.44 days and 10 days, respectively, whereas the mean length of ICU stay in survivors was 2.81 days. ESS correlated positively with the length of hospital and ICU stay among the survivors (rho=0.51 and rho=0.53, respectively). For a one-unit rise in ESS, the length of hospital stay and the length of ICU stay increased by 1.8 days and 0.71 days, respectively. Kaafarani et al. found that the median length of hospital stay was 11 days [10]. In a prospective study, Alburakan et al. found that the median length of hospital stay was four days (IQR: 2-9), and ESS was strongly correlated with length of hospital stay, with each unit increase in ESS resulting in a 2.7-day increase in hospital stay [18].

Using ROC analysis, the ESS in this study population showed a strong correlation with the 30-day mortality rate, with an AUROC of 0.895. Sangji et al., who are credited with developing the ESS, also documented an AUROC of 0.85 [8]. AlSowaiegh et al. performed a retrospective analysis of emergency surgeries across all surgical specialties. They found that ESS consistently showed a positive correlation with the 30-day mortality rate, with an AUROC curve ranging from 0.67 in cardiac surgeries to 0.97 in gynecologic surgeries [19]. This demonstrates the versatility and accuracy of ESS in predicting outcomes across various surgical specialties.

In our study population, the AUROC for ESS and 30-day complications was 0.875, demonstrating good diagnostic performance. A retrospective evaluation of ESS to predict complications by Nandan et al. found the AUROC for any 30-day complication to be 0.78 [14]. Other prospective studies also found the AUROC to lie in the range of 0.74-0.84 [7]. However, none of these studies mentioned the relationship to various grades of complications. The complications in our study population were graded as per the Clavien-Dindo classification. On comparison, we found that these different groups differed significantly in terms of ESS (χ2=87.686, p<0.001). We sought to determine the effect of ESS on the 30-day readmission rate. As there were only five readmissions in this study population, no significant relationship was found between ESS and the 30-day readmission rate.

Our study's findings underscore the potential of ESS as a powerful predictor of postoperative outcomes in emergency laparotomies. ESS can significantly enhance preoperative patient counseling and decision-making by providing an objective measure for outcome comparison. The findings highlighted by Havens et al. in their review also emphasize the significant potential of ESS in the context of emergency surgery [3].

Major limitations of our study were relatively small sample size and evaluation of ESS in patients undergoing emergency laparotomies alone. Its potential applicability to all emergency general surgeries and across different specialties presents a new domain for exploration. Additionally, investigating the effectiveness of ESS in predicting outcomes in non-operative patients could significantly influence patient management and decision-making. The unknown predictive power of ESS for long-term outcomes also calls for long-term follow-up studies.

## Conclusions

The ESS is an effective risk assessment tool for emergency laparotomies, consistently predicting postoperative outcomes in terms of mortality rate, complication rate, length of hospital stay, and length of ICU stay. It can be used in resource-limited settings to triage patients based on available resources, and also helps in benchmarking to improve post-operative outcomes for patients undergoing emergency laparotomy. ESS is a promising tool, and in the hands of able surgeons, it can become a vital cog in the management of patients undergoing emergency laparotomies.

Postoperative complications have a considerable impact on the quality of life and the cost borne by the patient. This can be overcome by benchmarking targets and comparing them with optimal outcomes. ESS comes in handy here by providing us with an objective measure of outcome comparison. The lacunae in the care provided can be identified, which helps in identifying areas for modifications and improvements to provide the best possible care to the patient.
